# Bullous Cutaneous Lesions in Pediatrics: A Diagnostic Challenge

**DOI:** 10.1177/00099228251375917

**Published:** 2025-09-25

**Authors:** Marcos Medina Bethencourt, Francisco José García Díaz, Claudia Andrade Díaz, Blanca Lanero Olivencia, Isabel Martínez Carapeto

**Affiliations:** 1 Departmen of Paediatrics, Hospital Universitario Virgen del Rocío, Sevilla, Spain

**Educational Objectives**

- Recognize the importance of differential diagnosis in pediatric patients with bullous cutaneous lesions.
- Understand the key clinical, histopathological, diagnostic, and therapeutic aspects of linear IgA dermatosis in children.

## Case Report

We present a case of a 3-year-old boy with no significant personal medical history who consulted the emergency department due to pruritic cutaneous lesions of 1 year’s duration. The lesions appeared in episodes, initially located on the buttocks and later spreading to the extensor surfaces of the limbs and perioral region. He remained afebrile throughout and had no general systemic involvement.

The patient had previously consulted several times, being diagnosed with impetigo and receiving multiple cycles of topical corticosteroids and antibiotics, with no improvement.

**Image 1.**
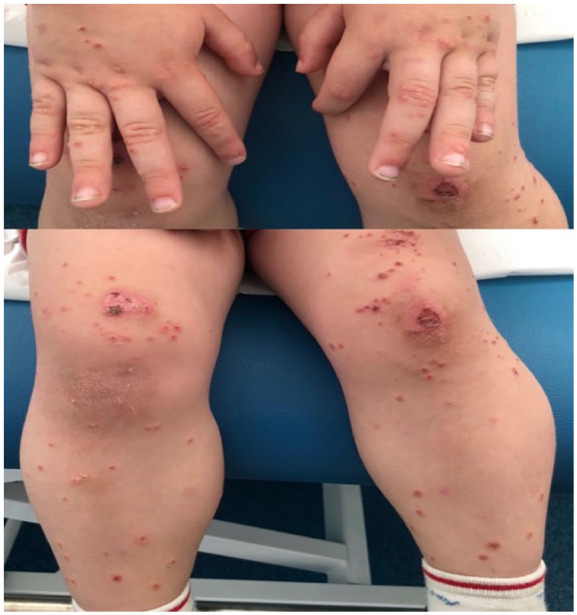
Tense vesicles with clear contents, excoriated and grouped, some with an ulcerated appearance, located on the dorsal surfaces of hands, knees, and pretibial region.

On examination, clear, excoriated and grouped vesicles were found periorally, on the elbows, hands, knees, pretibial area, and perianal region (Image 1). There was no mucosal involvement or other significant findings.

## Hospital Course

**Image 2.**
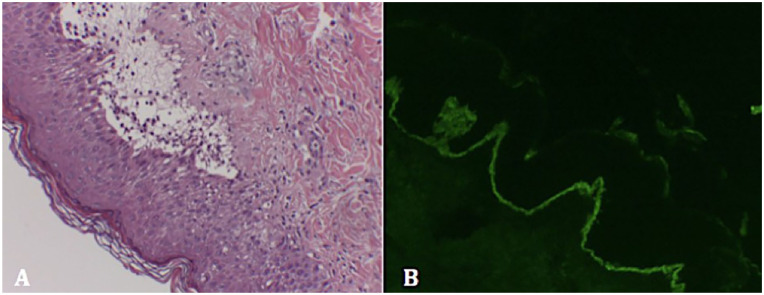
Punch skin biopsy. (A) Hematoxylin and eosin staining: subepidermal microabscesses are observed at the tips of epidermal papillae with accumulation of neutrophils and eosinophils, converging to form subepidermal blisters. (B) Direct immunofluorescence: linear deposit of IgA along the basal membrane.

Given the described findings and the slow progression of the patient’s condition, he was referred to the pediatric dermatology department, where a punch skin biopsy was performed. Microscopy revealed subepithelial blisters and an accumulation of neutrophils and eosinophils in the dermal papillae. Direct immunofluorescence showed a linear deposit of IgA at the dermal-epidermal junction (Image 2). A swab of wound exudate for varicella-zoster virus and herpes simplex virus types 1 and 2 DNA was negative. Blood tests showed no abnormalities except for anti-epidermal intercellular substance antibodies +1/40. The patient also had one of the HLA allele combinations associated with celiac disease, but anti-tissue transglutaminase antibodies were negative. Pending biopsy results, treatment with fusidic acid cream, methylprednisolone, astringent compresses, and hydroxyzine for itch control was initiated.

## Final Diagnosis

With the histopathological and immunofluorescence findings, the final diagnosis was linear IgA dermatosis (LAD). Treatment with dapsone at 1.5 mg/kg/d was started after excluding glucose-6-phosphate dehydrogenase (G6PD) deficiency. After 1 month, the dose was reduced due to improvement, but a relapse with new bullous lesions on the lower limbs necessitated resuming the initial dose. At 3 months, clinical improvement was evident, with only minimal perioral lesions that resolved with topical fusidic acid. Serial blood tests were performed to monitor for blood count and liver function abnormalities. After 15 months of dapsone treatment, the disease was in remission, and the medication was discontinued. The patient continued follow-up with dermatology, with no disease activity in the last 12 months.

## Discussion

Linear IgA dermatosis, also known as chronic bullous disease of childhood,^1,2^ is a rare autoimmune mucocutaneous disorder characterized by subepithelial vesicles and bullae.^3^ It has an incidence of 0.5-2.3 cases per million people/year^4^ and is the most common bullous disease in children.^5^ It has a bimodal age distribution, with a peak incidence in childhood (maximum at 4.5 years) and the sixth decade of life.^5,6^ In our case, the age of presentation is close to the peak incidence in children. There is no clear ethnic or gender predilection, though higher frequencies have been noted in South Africa, North Africa, and Asia.^7,8^

The exact pathophysiological mechanism is unknown.^8^ There is a strong association with human leukocyte antigens (HLA) (B8, CW7, and DR3, DQ2) and an allele of tumor necrosis factor alpha-2.^6^ The patient in our case was HLA-DQ2-positive. Both humoral and cellular responses are believed to be involved in pathogenesis.^3,5^ The humoral response includes the production of pathological IgA against basal membrane antigens, LAD-1, and LABD-97, which are part of the ectodomains of BP180 (collagen XVII). Autoantibodies against collagen VII, BP230, integrin α6β4, and laminin have also been described. The cellular response involves the presence of neutrophils and eosinophils that cause tissue damage and disruption of the basal membrane through cytokine release.^2,5,8^

The identification of HLA alleles commonly associated with celiac disease, such as HLA-DQ2 and HLA-DQB102:01, in this patient—despite negative celiac serology—raises important considerations regarding immunogenetic susceptibility in LAD. Recent studies have demonstrated that HLA-DQB102:0,^9^ a well-established celiac risk allele, is also found at increased frequency in patients with LAD, suggesting a potential shared immunogenetic background between these conditions. This overlap may reflect a broader predisposition to autoimmunity, as HLA class II alleles are known to confer risk for multiple autoimmune diseases, including type 1 diabetes, Graves disease, and systemic lupus erythematosus. The presence of these alleles in LAD, even in the absence of clinical or serological evidence of celiac disease, supports the hypothesis that certain HLA haplotypes may facilitate aberrant immune responses leading to cutaneous autoimmunity.^9,10^

Most cases are idiopathic, but potential triggers include medications, with vancomycin being the most frequent in adults, though less so in children.^6^ Other associations include β-lactams, nonsteroidal anti-inflammatory agents, and anticonvulsants.^1,7^ Less frequent associations include vaccinations (human papillomavirus vaccine), infections, skin trauma, autoimmune diseases (ulcerative colitis and systemic lupus erythematosus), and neoplasms (non-Hodgkin lymphoma and chronic lymphocytic leukemia).^2,11-13^ In some neonatal cases, maternal autoantibody transfer through breast milk has been described.^14^

Clinically, the disease presents with clear or serohematic vesicles and bullae on normal or erythematous skin.^5^ In children, these often group into rosettes with a central crust and peripheral bullae, creating the characteristic “strings of pearls.”^4,13,14^ Our patient’s lesions matched this description, although the rosette formation was absent, possibly due to prior topical corticosteroid and antibiotic treatment. Lesions typically localize to the perioral region, trunk, axillae, lower abdomen, and thighs but can also affect hands and feet.^7,8,11^ Oral and conjunctival mucosa involvement is possible but more common in adults. Lesions can cause varying degrees of itching.^2,3,5^

Diagnosis is based on clinical presentation, histopathology, and immunodiagnostic tests.^8^ Histopathology shows subepithelial blisters with an inflammatory infiltrate, primarily neutrophils, and sometimes eosinophils and lymphocytes.^5,8,15^ Diagnostic confirmation is achieved with direct immunofluorescence (DIF), indirect immunofluorescence, ELISA, immunoblot (Western blot), and electron microscopy.^1,5,16^ Direct immunofluorescence is the gold standard, showing linear IgA deposits along the basal membrane, sometimes with IgG, IgM, and complement C3.^3,8^

Differential diagnoses include herpetiform dermatitis, bullous pemphigoid, herpes simplex, bullous impetigo, erythema multiforme, and hereditary bullous epidermolysis. In cases without bullae, or with highly pruritic eruptions, atopic dermatitis and scabies may be considered.^2,3,13^ A comprehensive differential diagnosis was essential during the initial evaluation of this patient, given the broad spectrum of vesiculobullous diseases in childhood. The main entities considered included impetigo (particularly bullous impetigo), dermatitis herpetiformis, and bullous pemphigoid. Impetigo was initially suspected due to the presence of superficial blisters and erosions, but the lack of honey-colored crusts, negative bacterial cultures, and poor response to antibiotics argued against this diagnosis. Dermatitis herpetiformis was considered given the pruritic, grouped vesicles and distribution on extensor surfaces; however, DIF revealed linear, rather than granular, IgA deposition along the basement membrane, and there was no evidence of gluten-sensitive enteropathy. Bullous pemphigoid, although rare in children, was also evaluated; the absence of linear IgG and C3 deposition on DIF and the patient’s age made this diagnosis less likely. Ultimately, the diagnosis of LAD was established based on the clinical pattern of annular, grouped vesicles (“string of pearls”), the histopathological finding of subepidermal blisters with neutrophilic infiltrate, and the characteristic linear IgA deposition on DIF.

First-line treatment is dapsone, starting at 0.5 mg/kg with gradual increase to 2 to 3 mg/kg based on response and tolerability.^1,5,13^ The therapeutic effect can be seen within days.^8^ Despite good tolerability in most patients, potential severe side effects include methemoglobinemia, dose-dependent hemolysis, agranulocytosis, or peripheral neuropathy. The G6PD deficiency should be excluded before initiating treatment.^5,7,8,14^ Topical corticosteroids can be used as adjuncts. For treatment-resistant cases or when dapsone is intolerable, alternatives include oral corticosteroids, sulfapyridine, colchicine, intravenous immunoglobulin, or immunosuppressors like azathioprine, methotrexate, or mycophenolate mofetil.^1,5,8^ Newer drugs such as omalizumab, etanercept, or dupilumab have shown promising results.^1,14^

Prognosis is generally favorable, with disease resolution often occurring before puberty. However, severe morbidity and persistence into adulthood can occur. The average disease duration is 14 months, with approximately 64% of children achieving remission within the first 2 years.^5,11^

## Conclusion

Linear IgA dermatosis is a rare autoimmune disease predominantly affecting children, characterized by subepithelial bullae and linear IgA deposits in the basal membrane of skin and mucous membranes. The exact etiology remains unknown, but immunogenetic factors and external triggers such as medications are implicated. It is important to note that LAD does not follow a Mendelian inheritance pattern. Familial clustering is rare, and no monogenic mutations have been identified to date. The disease is typically an acquired autoimmune condition.

Diagnosis relies on clinical presentation, histopathology, and immunological tests, with direct immunofluorescence being the gold standard. It should always be considered in the differential diagnosis of bullous cutaneous lesions in pediatric patients.

Dapsone is the treatment of choice, often combined with topical or systemic corticosteroids. Its use may lead to potentially serious side effects such as methemoglobinemia and dose-dependent hemolysis, necessitating close monitoring and careful patient follow-up during treatment. For refractory cases or when dapsone is not tolerated, alternative therapies (eg, sulfapyridine, colchicine, intravenous immunoglobulin, azathioprine, methotrexate, mycophenolate mofetil) should be considered, emphasizing the need for an individualized treatment approach.

While LAD typically has a favorable prognosis with spontaneous resolution in many cases, long-term follow-up is essential to detect potential recurrences or late complications. Early diagnosis and appropriate treatment are crucial for improving outcomes.

## Author Contributions

Marcos Medina Bethencourt, Francisco José García Díaz, Claudia Andrade Díaz, Blanca Lanero Olivencia and Isabel Martínez Carapeto: Contributed to conception and design; Contributed to analysis; Drafted the manuscript; Critically revised the manuscript; Gave final approval; Agrees to be accountable for all aspects of work ensuring integrity and accuracy.
